# Measuring women’s agency in family planning: the conceptual and structural factors in the way

**DOI:** 10.1080/26410397.2022.2062161

**Published:** 2022-06-01

**Authors:** Nandita Bhan, Anita Raj, Edwin E. Thomas, Priya Nanda, The FP-Gender Measurement Group

**Affiliations:** a Research Scientist, Center on Gender Equity and Health, University of California San Diego, School of Medicine, La Jolla, CA, USA.; b Tata Chancellor Professor of Society and Health, Department of Medicine; Professor of Education Studies, Division of Social Sciences; Director, Center on Gender Equity and Health, University of California San Diego, School of Medicine, La Jolla, CA, USA; c Research Coordinator, Center on Gender Equity and Health, University of California San Diego, School of Medicine, La Jolla, CA, USA; d Senior Program Officer, India Country Office, Bill & Melinda Gates Foundation, Seattle, USA

**Keywords:** women’s agency, sexual and reproductive health, reproductive autonomy, gender equity

In 1994, the International Conference on Population and Development (ICPD) called for centralising women’s agency within reproductive health research and programmes. This shift was made possible by years of feminist scholarship and activism within population and development programmes. Even as global development goals have acknowledged the importance of women’s education and rights in moving the needle for reproductive health access, ICPD recommendations have not been fully integrated into health programmes. As a result, almost 30 years later, despite evidence on the importance of women’s empowerment for health seeking^1–5^, family planning programmes continue to see contraception use and targets as the predominant goals for nations and communities, and contraceptive use as a marker of women’s choice in family planning. Barriers to centralising women’s agency in sexual and reproductive health programmes have included conceptual and methodological challenges in measuring women’s empowerment and choice, particularly women’s agency in influencing family planning behaviours and service use, within the theory of change and evaluations.

*The lag in prioritising women’s agency over contraceptive targets may be due in part to the lack of consensus in the meaning and measurement of agency and choice specific to sexual and reproductive health.* Shared consensus and vision are needed both at the *conceptual level* (definitions and components, theorising connections to outcomes and developing survey measures) and in relation to *structural dimensions in the field* (the flow of learnings and resources between researchers and implementers, and the space given to women’s voices within research and programmes).

## What do we know about women’s agency? What gaps in knowledge persist?

An evidence review^6^ of women’s agency measures used in family planning research and programmes shows that the field uses a diversity of constructs to define women’s power, voice and choice within the contraceptive user journey, including *women’s autonomy*, *family planning self-efficacy*, *reproductive coercion*, *quality of care*, *male engagement* and *attitudes related to contraceptive use.* However, the review also reveals several theoretical as well as empirical challenges that continue to plague conceptualisation and empirical validation of measures. Measures of women’s agency can be summarised into *four typologies in family planning programmes*:

- ***Constructs and measures that are well-understood or accepted*** among scholars as best evidence measures: these have demonstrated validity along with ease of use, brevity or clarity, but need wider recognition and use in the field (e.g. *decision-making agency*^7^ and *reproductive coercion*^8^). New measures, for instance, like decision-making agency, go beyond who made the final decision and capture the decision-making process, how perspectives are shared and valued, and agreed upon^7^. Similarly, the reproductive coercion measure provides an understanding of resistance faced by women from their partners in exercising their contraceptive choices^8^.
- ***Constructs and measures that are being tested across diverse contexts or populations:*** (e.g. a *family planning norms*^9^ measure that demonstrates validity in one context, but needs further adaptation and testing to assess relationships with family planning outcomes). This measure allows us to understand the enabling environment driving contraceptive choices, but can be context-specific in their content and use.
- ***Constructs for which measures exist and need adaptation, particularly simplification in construct or measure length for ease of use in programmes:*** **For example, lengthy proven** measures such as *family planning self-efficacy*^10^ and *contraception stigma*^11^ could be created as short-form measures for integration into family planning programme surveys and into monitoring and evaluation systems.
- ***Constructs for which promising measures need further conceptualisation and theorisation, more formative research and psychometric testing:* These include** ***fertility preferences*** (**i.e.** intention, choices, timing, preferences around completion of family size, women’s or couple’s ability to conceive); *negotiation* and *decision-making* including *reproductive bargaining* (e.g. what a woman may give up to get her husband’s acquiescence to her reproductive choice); *norms related to fertility planning and contraceptive use*; *couple communication* on sex; *method switching* and *discontinuation*; and *stigma* including *backlash* (e.g. abuse or alienation from husband or family as a consequence of her contraceptive choices).

In addition to these typologies of constructs and measures of women’s agency in family planning, there is also variation in and under-representation of key populations and constituencies including adolescents, young unmarried women and men, zero- and low-parity women, non-heterosexual/non-cisnormative couples/individuals, and couples who do not desire children, in family planning research and programmes. Family planning surveys and programmes at present focus disproportionately on married populations, more specifically women, reinforcing family size goals and contraceptive targets instead of recognising the circumstances or contexts wherein women make fertility and family planning choices. Youth, in particular, and their sexual activity prior to marriage often continue to be stigmatised and the needs for and barriers to health services among youth remain unrecognised and unaddressed. Men have also not received adequate focus in family planning programmes, unless they are husbands, reinforcing norms that women, and not men, are responsible for contraceptive use. Also under-represented has been an understanding of women’s agency as expressed by non-use, non-desire for contraception or ambivalence towards contraception use. The under-representation of these groups and themes in family planning research and programmes furthers marginalisation and creates wider gender asymmetries in contraceptive access.

## What are the conceptual barriers to measuring women’s agency in family planning?

While feminist and women’s rights scholars have advanced our understanding of women’s empowerment for health^1–5^, these learnings have not been adequately extended to measuring women’s agency in family planning programmes. This limitation may be due to two reasons.

First, in family planning, researchers have often referred to a diverse range of concepts to measure women’s (reproductive) agency without explicitly locating them within the theory of change in family planning programmes. This lack of conceptualisation and of drawing linkages to outcomes is also noted in the over and interchangeable use of generalised measures of self-efficacy or household decision-making as proxy measures of women’s agency in family planning. While these measures may be valuable in themselves and readily available through large-scale surveys, they lack items that capture family planning context or relevance. For instance, generalised measures of empowerment may not draw on women’s ability to voice their choices pertaining to childbearing, contraception, ideal number of children as well as themes such as bodily autonomy, women’s social position within their family or community and aspirations regarding building a family.

*The field needs to critically examine the existing science on women’s agency measures, focused on the individual or the interactional, to understand in which ways current conceptualisations draw from theoretical and empirical understandings of family planning and gender, and how they are informing programmes around contraception choice, uptake and use.* Measurement innovations such as *decision-making agency*^7^ have questioned the present focus in survey measures on *who decides* and have extended them to include *what matters* to women, whether women share their preferences and feel valued, and are satisfied with their participation and the decision.

The scholarship on measuring women’s agency is also increasingly recognising constructs that operate at the couple, household and community levels, reflecting agency as bargaining and negotiations with one’s partner, experiencing or resisting social pressures to bear children, and encountering support or coercion in contraception use or non-use. The interactions of these constructs with racial, ethnic and other social inequities also bring to light the intersectionalities and variations in voice, choice and participation.

A second barrier to measurement of women’s agency has been the gap between researchers and implementers, despite the need for research and implementation to be interlinked and iterative. This barrier has led to a dichotomy wherein, on the one hand, greater embedding of measures within family planning programmes is needed to provide empirical evidence on women’s agency measures; on the other hand, programme implementers very often do not have access to new or emerging evidence with respect to tested or validated measures. This silo-ed nature of the field has also led to disciplinary tensions between the fields of gender and public health, with the former emphasising contraception choice (to use or not use) as the goal, while the latter is prioritising health-related outcomes of contraception use. In our view, even as these silos get reconciled, it continues to be important to emphasise gender and rights frameworks within public health programmes so as to value women’s satisfaction, dissatisfaction and even ambivalence with the choices available to them in family planning programmes. At the same time, newly developed and viable measures of women’s agency in family planning^7–9^ must be palatable, accessible, appropriate for the context and easy to use for family planning implementers.

In family planning, unlike some other domains of women’s empowerment research, there is recognition that measures of reproductive agency^12,13^ can be attentive to issues at both the individual as well as couple levels, depending on the context. This seeming dichotomy can also be resolved through dialogue between family planning researchers and implementers on effective integration of gendered constructs within family planning programmes. Similarly, striking a balance between context specificity and cross-national comparability is an important challenge for measurement. This balance requires communication and partnerships between implementers and a wider stakeholder group for greater harmonisation of measures across contexts and for use within family planning programmes.

## How can we improve measurement of women’s agency in family planning to meet community needs and strengthen family planning programmes?

Despite a vibrant group of scholars, researchers and practitioners advocating for the goal of centralising women’s agency in family planning, programmes and evaluations continue to lack a diverse representation of disciplines and geographies. While feminist scholarship over the decades has often been led by contributions from the global South, the architecture of knowledge, evidence and the flow of resources related to key sexual and reproductive health and rights issues continues to be driven from the global North to the global South. Scholarship and implementation research in the most affected nations and populations has often been led by those not in these nations and of these populations. More inclusive approaches to research generation are needed in family planning that can build a diverse and representative pipeline of scholars for future research in these areas and to bring forward the learnings on women’s agency from a greater diversity of contexts.

To overcome some of these disciplinary and data bottlenecks, the field may need three sets of synergies. *First*, there is a need to better leverage existing and routine data collection opportunities, particularly in low- and middle-income countries, including national health management information systems and routine surveys that will allow practitioners to embed and examine women’s agency and choice within family planning programmes. Examining these issues through routine data systems can inform policy and strategic partnerships in real time.

*Second*, the field often derives learnings on women’s agency from large cross-national surveys. There is an increasing recognition of the need to support these surveys with other methodologies including qualitative and mixed-method observational studies, and evaluations. Emerging methodologies including vignettes, digital platforms, big data and phone surveys may also be useful in filling key data gaps and give voice to community needs and the lived realities of women in innovative ways. Creative use of digital platforms can allow practitioners to engage more deeply with questions related to access, women’s choice and agency as well as the user experience.

*Finally*, despite the contribution of gender rights-based scholarship and landmark ideas from the ICPD in driving the field of sexual and reproductive health, the present field of family planning programmes needs more cross-disciplinary collaborations. Investments to increase conversations across disciplines and between scholars and practitioners across fields (e.g. community development, human rights, child marriage, trafficking and violence prevention) can improve our insight into what works to improve women’s access to and agency in family planning.

Without engaging in cross-disciplinary research partnerships, family planning scholars and implementers will find it challenging to present to donors and governments the evidence needed for mainstreaming women’s agency in family planning programmes.

## What is the way ahead?

In summary, while the field of family planning has advanced considerably, there is an urgent need to shift from approaches that focus on contraceptive outcomes as a proxy for women’s agency to prioritising women’s reproductive choice and agency as outcomes in themselves. This must draw from cross-disciplinary scholarship across the fields of family planning or public health, as well as from sociology, women’s studies and gender studies^14,15^. While several existing and emerging measures of women’s agency are promising, family planning researchers need to make a better case with rigorous evidence that demonstrates the importance of this work for women’s reproductive goals as well as to expand choice within family planning programmes. Investments that centralise women’s agency within family planning programmes may hold the key to strengthening family planning programmes, address unmet sexual and reproductive health needs and deliver effective family planning services to communities.

## The FP-Gender Measurement Group (in alphabetical order)

Fred Arnold, PhD, ICF
Sarah Averbach, MD, Center on Gender Equity and Health, UC San Diego
Sarah Baird, PhD, George Washington University
Cari Jo Clark, ScD, Emory University
Kathryn Conn, PhD, The Women’s Storytelling Salon
Elizabeth Costenbader, PhD, FHI360
Michele R Decker, ScD, Johns Hopkins University
Christine Dehlendorf, MD, University of California, San Francisco
Leanne Dougherty, PhD, Population Council
Anvita Dixit, PhD, Center on Gender Equity and Health, UC San Diego
Jeff Edmeades, PhD, Avenir Health and the DHS Program
Anna Mia Ekström, MD, Karolinska Institute
Laura Hinson, PhD, International Center for Research on Women
Kelsey Holt, ScD, University of California, San Francisco
Sandra Jordan, MA, What Works Association & Independent Consultant
Celia Karp, PhD, Johns Hopkins University
Rebecka Lundgren, PhD, Center on Gender Equity and Health, UC San Diego
Ann M Moore, PhD, Guttmacher Institute
Caroline Moreau, MD, Johns Hopkins University
Nandini Oomman, PhD, The Women’s Storytelling Salon
Elisabeth Rottach, MA, Palladium
Dominick Shattuck, PhD, Johns Hopkins Center for Communication Programs
Jay Silverman, PhD, Center on Gender Equity and Health, UC San Diego
Ilene S Speizer, PhD, University of North Carolina at Chapel Hill
Carol Underwood, PhD, Johns Hopkins University
Shannon N Wood, PhD, Johns Hopkins University
Jennifer Yore, MPH, Center on Gender Equity and Health, UC San Diego
Kathryn M Yount, PhD, Emory University
